# 104 Indocyanine Green Fluorescence Differentiates Necrotic Cells from Healthy and Apoptotic Cells

**DOI:** 10.1093/jbcr/irad045.077

**Published:** 2023-05-15

**Authors:** Jocelyn Zajac, Sameeha Hassan, Angela Gibson, Aiping Liu

**Affiliations:** University of Wisconsin-Madison, Madison, Wisconsin; University of Wisconsin-Madison, Madison, Wisconsin; University of Wisconsin School of Medicine and Public Health, Madison, Wisconsin; University of Wisconsin-Madison, Madison, Wisconsin; University of Wisconsin-Madison, Madison, Wisconsin; University of Wisconsin-Madison, Madison, Wisconsin; University of Wisconsin School of Medicine and Public Health, Madison, Wisconsin; University of Wisconsin-Madison, Madison, Wisconsin; University of Wisconsin-Madison, Madison, Wisconsin; University of Wisconsin-Madison, Madison, Wisconsin; University of Wisconsin School of Medicine and Public Health, Madison, Wisconsin; University of Wisconsin-Madison, Madison, Wisconsin; University of Wisconsin-Madison, Madison, Wisconsin; University of Wisconsin-Madison, Madison, Wisconsin; University of Wisconsin School of Medicine and Public Health, Madison, Wisconsin; University of Wisconsin-Madison, Madison, Wisconsin

## Abstract

**Introduction:**

No objective technique exists to distinguish necrotic from viable tissue, risking over-excision in burns and loss of wound healing potential. Using delayed fluorescence imaging of indocyanine green (ICG), a method called second window ICG (SWIG), we have shown that high dose ICG persists in burn wounds in both animals and patients 24 hours after infusion. The necrotic avidity of ICG is proposed as the underlying mechanism, although inflammation also likely has a role. The objective of this study is to examine the hypothesis at both the tissue and cellular levels.

**Methods:**

To examine the localization of ICG in burn tissue, 4-mm biopsies taken from burn patients who underwent SWIG were embedded and sequentially sectioned for H&E (tissue architecture) and LDH (tissue viability) and ICG fluorescence imaging. To examine whether ICG preferentially binds to necrotic cells, primary skin cells (fibroblasts or keratinocytes) were subjected to thermal injury at 65ºC for 0 (untreated), 2.5 or 5 minutes. The cells were subsequently incubated with ICG at concentrations ranging from 0.5 to 5 μg/mL at 37ºC for 30 minutes. A necrosis and apoptosis detection kit with Yo-Pro™-1 and propidium iodide (PI) was used to differentiate live, apoptotic and necrotic cell populations in flow cytometry. The ICG florescence intensity of each cell population was measured with flow cytometry.

**Results:**

The fluorescence microscopic imaging revealed that ICG intensity was most intense in the superficial severely thermally-injured tissue and gradually decreased with depth. This zone of high ICG intensity was co-localized with high cellularity (shown in H&E) consisting of a mixture of nonviable skin cells (shown in LDH) and inflammatory infiltrates. Using flow cytometry, the optimal concentration of ICG with the highest ICG florescence median intensity (FMI) and the best separation of live, apoptotic and necrotic cell populations was 2.5 μg/mL, which was used for subsequent flow cytometry experiments. For both fibroblasts and keratinocytes, the ICG FMI was significantly higher in the necrotic cell population than that of either the live or apoptotic cell population, suggesting higher affinity of ICG to necrotic cells. The ICG FMI of necrotic cells with longer heat exposure (5 minutes) was significantly higher than that of necrotic cells with shorter heat exposure (2.5 minutes), suggesting that the binding sites for ICG increase with the degree of thermal injury.

**Conclusions:**

This study demonstrates the necrotic avidity of ICG in burned skin tissue and two primary skin cell types. Further studies are needed to study the ICG binding characteristics with inflammatory cell populations and single cell suspensions dissociated from human burn tissue.

**Applicability of Research to Practice:**

This, along with our pilot study of SWIG in patients, supports the feasibility of SWIG in guiding surgical decision-making in burn excision by defining thresholds of fluorescence intensity values.

